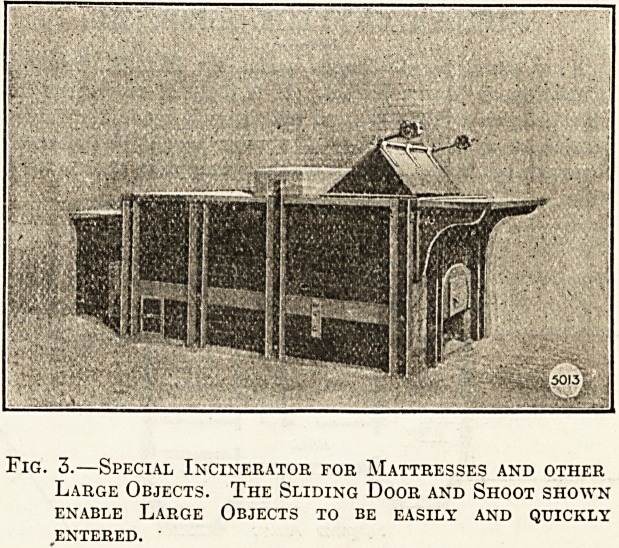# An Incinerator for Infected Materials

**Published:** 1909-08-14

**Authors:** 


### HOSPITAL ADMINISTRATION.

### CONSTRUCTION AND ECONOMICS.

### AN INCINERATOR FOR INFECTED MATERIALS.

All hospitals, sanitary institutions, and the like, have a serious problem in the safe disposal of dressings, infected clothing, mattresses, and sometimes of fæcal matter. In the old days they were too often allowed to go the way of other refuse. All that could be was washed down the drains, and the rest was either buried or carted off with the refuse of the district.

Fig. 1.—Front View of Ordinary Form of Incinerator. The Fuel Door is shown in Front and the Sliding Doors for Refuse are Both at the Back.

Modern sanitation will not allow of this. Burying is often out of the question, if it were not forbidden for sanitary reasons. Hospitals are placed where they can be of the greatest service, and that is usually in the midst of a busy part of one or other of our great towns, and the space available for the purpose would be soon filled up. The materials referred to are not worth the trouble and expense of disinfecting, and there is, in addition, a lot

Fig. 2.—Back View of Ordinary Form of Incinerator showing the Auxiliary Furnace.

of garbage and other matter proceeding from a hospital that must be got rid of in some harmless manner. Modern science points to one method as combining the necessary qualifications for disposal of matter of the kind, and that is by burning. Burnt things do not take up room, like buried things do, and if the burning is properly carried out, there should be no chance of the spread of infection.

In order that burning shall be properly and economically carried out, certain conditions are necessary. The apparatus employed should be easily handled by the ordinary hospital staff, and should not readily get out of order. It should be so arranged that the infected articles can be easily passed into it, and they should not require any special preparation. It should be capable of dealing with anything that it is found convenient to cremate, at any time of the day or night, and of any size within certain limits. In addition it should ensure complete cremation, not only of the substances themselves but of the primary products of combustion. The death point of nearly all the bacilli of diseases, as is well known, is comparatively low, very much below the temperature of an ordinary red coal or coke fire; but in order to make sure that the hot gases issuing from the apparatus employed to burn the infected material shall not communicate disease or be a nuisance to the neighbourhood, it is necessary, according to modern research, that the primary products of combustion, the substances produced by what may be termed the first burning of the articles, should be

Fig. 3.—Special Incinerator for Mattresses and other Large Objects. The Sliding Door and Shoot shown enable Large Objects to be easily and quickly entered.

subjected to a temperature of from 1500° to 2000° F., in order that the final products may issue from the chimney in a harmless condition. If this is not done, though the hot gases passing from the chimney may not contain any disease germs, what they carry may form a bed for germs existing in the atmosphere around, with the result that an effect may be produced nearly as harmful as that produced by the older methods. Further, the apparatus employed for the purpose must not take up much room. Modern hospitals, sanatoria, etc., have so many demands upon their space that they cannot afford much room even for such an important apparatus as this.

In the accompanying illustrations we show apparatus of this kind, made by Messrs. Manlove Alliott and Co., of Nottingham, who have made a special study of problems of this kind. The apparatus consists of two chambers—one in which the primary combustion takes place, and the other in which the products of the primary combustion are themselves subject to the high temperatures mentioned. The first chamber is a furnace arranged to receive any kind of material that may be delivered to it, and to consume it quickly. Figs. 1 and 2 show the ordinary form of "Incinerator," as the firm have named the apparatus, and fig. 3 one specially designed to take mattresses and similar large objects. As will be seen, there is a sliding door at the back of the top of the furnace, through which the articles to be burnt are entered, the door being immediately closed. The articles to be burnt fall on to a drying plate under the sliding door, and thence pass into the furnace proper. In the apparatus shown in fig. 3 there is a shoot, as shown, extending the whole length of the furnace, so that large objects can be entered. There is also a door at the front through which fuel is entered, and through which articles can also be pushed. This is convenient for small articles. There is a small auxiliary furnace at the back, shown clearly in fig. 2, which is fed with cinders from the fire grates, or any cheap fuel that is handy, and the primary products of combustion pass through this furnace, where they are subjected to the high temperatures necessary. The "Incinerator" is made in various sizes, and for every kind and size of hospital or sanatorium. It is made in portable form, as well as to be fixed inside the building, and is claimed to be easily worked by the hospital staff. The final products of combustion escape to the general chimney quite harmlessly.

## Figures and Tables

**Fig. 1. f1:**
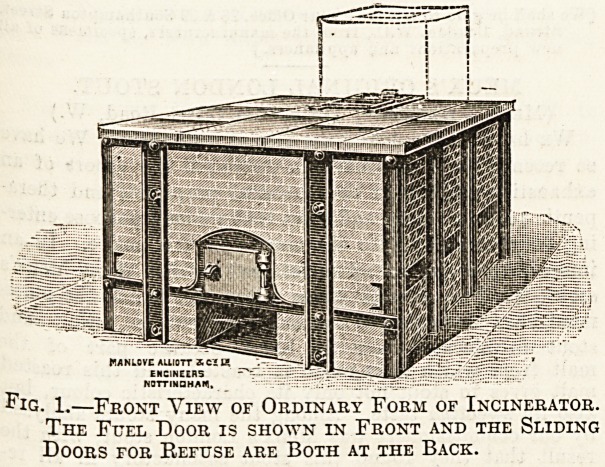


**Fig. 2. f2:**
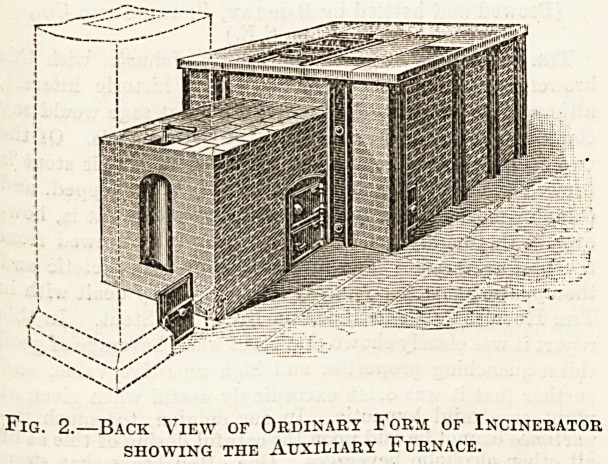


**Fig. 3. f3:**